# The Evolving Treatment Landscape of Advanced Renal Cell Carcinoma in Patients Progressing after VEGF Inhibition

**DOI:** 10.15586/jkcvhl.2017.69

**Published:** 2017-05-11

**Authors:** Pedro C. Barata, Moshe C. Ornstein, Jorge A. Garcia

**Affiliations:** 1 Department of Hematology and Medical Oncology, Cleveland Clinic Taussig Cancer Institute, Cleveland, OH, USA; 2 Department of Urology, Glickman Urological and Kidney Institute, Cleveland Clinic, Cleveland, OH, USA

**Keywords:** advanced renal cell carcinoma, immunotherapy, mTOR inhibitors, PD-1/PD-L1, sequential therapy, VEGF inhibitors

## Abstract

Despite significant changes in the therapeutic landscape of renal cell carcinoma, the majority of patients with metastatic disease eventually progress after first-line treatment with vascular endothelial growth factor receptors (VEGFR) tyrosine kinase inhibitor (TKI) therapy. Understanding existing data on subsequent therapies is crucial to define an optimal treatment sequence following first-line failure. This review examines the data supporting currently approved agents in this setting and provides a framework for decision-making regarding treatment sequencing beyond first-line therapy with VEGFR TKIs.

## Introduction

Significant changes in the therapeutic landscape of renal cell carcinoma (RCC) have drastically improved the outcome of patients with advanced RCC. Since the approval of high-dose IL2 (HD IL-2) in the mid-1990s (1, 2), more than eight new agents with novel mechanisms of action (MOA) are now available (3). These include seven VEGF inhibitors (4), two mammalian target of rapamycin (mTOR) inhibitors, and a novel immune checkpoint inhibitor (5). Despite this dramatic progress, complete and durable responses to these agents are rare, and eventual resistance to each agent is nearly universal.

The endothelial growth factor receptors (VEGFR) tyrosine kinase inhibitors (TKIs) sunitinib and pazopanib are approved for the vast majority of patients with untreated advanced RCC and are the standard of care in this setting (6, 7). Temsirolimus, a novel mTOR inhibitor, is the standard of care for untreated patients with multiple risk factors (poor-risk group). Drug intolerance and disease progression are the most common reasons for drug discontinuation, although the mechanisms of disease resistance are poorly defined. Unfortunately, despite the advent of the precision medicine and tumor genomics era, current treatment recommendations for advanced RCC beyond first-line treatment are largely driven by generally accepted consensus guidelines, side effect profiles, and quality of life (QOL) interests. Both VEGF and mTOR inhibition have demonstrated effectiveness in the second-line setting and beyond. However, the appropriate patient phenotype, ideal sequence, timing, and duration of therapy remain unknown.

In the setting of disease progression on VEGF-directed therapy, multi-kinase inhibition has become an attractive therapeutic option with agents such as cabozantinib, a VEGFR, MET, and AXL inhibitor (8). Similarly, the role of immunotherapy has also regained momentum with the recognition that checkpoint inhibition can lead to survival improvement as demonstrated with the use of the PD1 inhibitor nivolumab in the second-line RCC setting (9). Given a plethora of treatment options with various MOAs, understanding the data from existing trials in the refractory patient population is crucial to define the best management for patients. This review examines the data supporting currently approved agents in this setting and provides a framework for decisions regarding sequencing of therapy following first-line VEGF TKI therapy.

## Existing Evidence in Second-Line and Beyond

### REnal Cell cancer treatment with Oral RAD001 given Daily (RECORD-1)

Everolimus is an oral mTOR inhibitor, a key molecule that mediates cellular growth, proliferation, cellular metabolism, and angiogenesis (10). It exerts its function by inhibiting the intracellular protein FKBP-12 resulting in the inhibition of pS6 and 4EBP1 (11). RECORD-1 was a randomized, placebo-controlled international phase III trial that evaluated the efficacy of everolimus or placebo in over 400 patients with advanced RCC whose disease had progressed on VEGFR TKIs (12). The vast majority of patients had advanced RCC with a clear cell component and had disease progression on or within 6 months of stopping treatment with either sunitinb or sorafenib or both. Prior therapy with cytokines or bevacizumab was allowed. Patients were randomized in a 2:1 fashion to receive either everolimus 10 mg PO or daily placebo. The primary endpoint of this trial was progression-free survival (PFS) (Table 1). Secondary endpoints included overall response rate (ORR), safety, overall survival (OS), disease-related symptoms, and QOL. More than two-thirds of the patients on study were male and approximately 60% of them had a Karnofsky Performance Status (KPS) of 80%–90%. According to Memorial Sloan Kettering Cancer Center (MSKCC) risk score (13), more than 85% of patients in the trial were either good or with intermediate-risk disease. Although most patients had received one TKI previous to therapy (sunitinb 46% and sorafenib 28%), approximately 26% of them in both arms had received both oral agents and 9% also had received the VEGF monoclonal antibody bevacizumab.

**Table 1:** Second-line trials (INTORSECT/RECORD 1/AXIS).

	Temsirolimus	Everolimus	Axitinib
**MSKCC risk good/int/poor**	19/69/12	29/57/15	28/37/33
**Comparator**	Sorafenib	Placebo	Sorafenib
**Endpoint**	PFS	PFS	PFS
**ORR, %**	8	1	19
**PFS, months**	4.3	4.0	6.7
**OS, months**	12.3	14.8	20.1

MSKCC, Memorial Sloan Kettering Cancer Center; ORR, overall response rate; OS, overall survival; PFS, progression-free survival.

PFS assessed by independent central review was significantly longer for those receiving everolimus compared to those on placebo (4.0 vs. 1.9 months, respectively; HR 0.31, 95% CI 0.24–0.41, p < 0.0001). The PFS benefit was observed and maintained across pre-defined sensitivity and subgroup analyses. The Response Evaluation Criteria In Solid Tumors (RECIST)-defined (14) ORR was 1% for everolimus and 0% for placebo; however, a significant number of patients on everolimus (63%) achieved stable disease (SD) when compared to placebo (32%). The most common adverse events (AEs) observed on this study were stomatitis, rash, fatigue, asthenia, and diarrhea. The incidence of grade (G) 3 and 4 AEs was low. Despite this, treatment with everolimus led to a greater number of G3 and G4 stomatitis, infections, and non-infectious pneumonitis. Likewise, laboratory abnormalities including hyperglycemia, hypercholesterolemia, lymphopenia, and hypophosphatemia were observed more frequently on those patients receiving everolimus. Drug discontinuation or secondary drug-related AEs were observed in 10% of patients on everolimus and 4% on placebo. Similarly, dose modifications were required in 34% of patients receiving everolimus versus 15% on placebo. An updated report of this trial indicated that the median PFS was 4.9 months (everolimus) versus 1.9 months (placebo) (hazard ratio [HR], 0.33; P < 0.001) by independent central review (15). The median OS was 14.8 months (everolimus) versus 14.4 months (placebo) (HR, 0.87; P = 0.162), with 80% of patients in the placebo arm eventually crossed over to everolimus.

### Axitinib versus sorafenib (AXIS)

The phase III AXIS study evaluated the clinical efficacy and safety of axitinib versus sorafenib as a second-line therapy (16). Axitinib is a potent second-generation oral inhibitor of VEGFR1–3 with a relative potency of 50–450 times greater than that of the first-generation VEGF inhibitors (17). Another attractive feature of this agent is its lack of off-target effects and thus a very predictable AE profile (18). The primary endpoint of AXIS was PFS (Table 1). Secondary endpoints were similar to those of RECORD-1 and included ORR, safety, and a composite endpoint of duration of response and time to clinical deterioration. Over 720 patients whose disease had progressed on one previous systemic therapy (cytokines, mTOR inhibitors, and VEGF inhibitors with the exception of axitinib and sorafenib) for RCC were randomized 1:1 to receive either axitinib or sorafenib. Axitinib was administered as 5 mg PO twice daily (BID), and dose escalation to 7 mg and 10 mg PO BID was allowed for patients not experiencing G2 AEs. Sorafenib was administered as 400 mg PO BID. Pre-stratification according to Eastern Cooperative Oncology Group (ECOG) performance status (PS) 0 or 1 and type of previous treatment was also completed. Among the patients enrolled in the trial, 54% had received previous therapy with sunitinib, 35% cytokines, 8% bevacizumab, and 3% temsirolimus. Using MSKCC prognostic classification (13), 33% of patients were grouped under poor-risk. The median PFS was 6.7 months for axitinib and 4.7 months for sorafenib (HR 0.67, 95% CI 0.54–0.81, one-sided p < 0.001). When evaluating PFS based on previous therapy, for patients who had received previous cytokine-only therapy, the median PFS was 12.2 months for axitinib and 8.2 months for sorafenib (HR 0.51, 95% CI 0.37–0.68, p < 0.0001) (19). The median PFS for those who had received sunitinib was 6.5 months for axitinib and 4.4 months for sorafenib (HR 0.72, 95% CI 0.57–0.90, p = 0.002). The ORR observed for the entire patient population was 19% for axitinib and 9% for sorafenib. Recently, a post hoc analysis to determine the efficacy of axitinib and sorafenib based on response to previous therapy demonstrated that response to prior therapy does not predict for subsequent response to either agent (20). This study, however, demonstrated that longer response to first-line therapy generally yields a better outcome with second-line therapy (20). The most common AEs observed on axitinib included diarrhea, hypertension (HTN), nausea, dysphonia, and hypothyroidism. Skin rash, palmar–plantar erythrodysesthesia (PPE), and alopecia were more common with sorafenib therapy. Similarly, the most common G3 and G4 AEs reported include HTN, diarrhea and fatigue with axitinib and PPE, hypophosphatemia, elevated lipase, and HTN with sorafenib.

Recently, Motzer et al. (19) reported the analysis on PFS and OS from this study. The median OS was 20.1 months with axitinib and 19.2 months with sorafenib (HR 0.97, 95% CI 0.80–1.17, p = 0.3744). Similarly, the median investigator-assessed PFS was 8.3 months for axitinib and 5.7 months for sorafenib (HR 0.66, 95% CI 0.55–0.78, p < 0.0001). HTN was identified as a prognostic factor for survival in patients treated with axitinib (20.7 vs. 12.9 months, p = 0.012) and sorafenib (20.2 vs. 14.8 months, p = 0.002). In this updated analysis, AEs were similar to those reported initially.

### Investigating Torisel as second-line therapy (INTORSECT)

Although temsirolimus has been approved as first-line treatment for untreated advanced RCC patients with multiple risk factors based on the OS benefit reported in the global AARC trial (21), its clinical efficacy in the second-line setting was evaluated in the INTORSECT trial, a randomized international open-label, multicenter phase III study evaluating sorafenib and temsirolimus in patients whose disease progressed on sunitinib (Table 1) (22). A total of 512 eligible patients had histologically confirmed advanced RCC (any histology) with RECIST-defined (14) disease progression while receiving first-line therapy with sunitinib. All patients must have previously received at least 4 weeks of sunitinib-based therapy independent of dose or schema. Although patients with clinical progression were allowed to participate in the study, those who discontinued sunitinib secondary to AEs were not allowed to participate. Enrolled patients were randomized 1:1 and received either intravenous (IV) temsirolimus 25 mg once weekly or oral sorafenib 400 mg twice *per* day. Patients were stratified based on the standard disease features for advanced RCC studies. The primary endpoint was PFS with secondary endpoints that included ORR, OS, and safety. With a median follow-up of 9.2 months, the median PFS was 4.3 months for temsirolimus and 3.9 months for sorafenib (HR: 0.87; 95% CI 0.71–1.07; two-sided p = 0.19). The confirmed independent review ORR was 8% and this was similar in both arms. The median OS was significantly longer with temsirolimus than with sorafenib with 12.3 versus 16.6 months, respectively (HR1.31; 95% CI 1.05–1.63; two-sided p = 0.01). No new safety signals of concern were identified. In fact, the same proportion of patients experienced at least one G3 AE (70% with temsirolimus and 69% with sorafenib). G3 anemia and hyperglycemia were more common with temsirolimus (9% vs. 3% and 8% vs. 2%, respectively). Similarly, G3 PPE was more frequent with sorafenib (0% vs. 15%). AEs resulting in dose reductions were observed in 16% of the patients receiving temsirolimus and 33% on sorafenib, respectively. Although the lack of correlation between PFS and OS in this trial is not well understood, INTORSECT was the first study in the second-line setting demonstrating an OS benefit for patients with advanced RCC. The results, however, do not suggest that sorafenib is the second-line agent of choice, rather solidified the hypothesis that angiogenic escape is in fact one of the mechanisms resistant to first-line VEGFR inhibition, and thus, the possibility that sequential VEGF-based therapy is a logical treatment strategy in patients with advanced RCC.

### Global oncologic leanings for dovitinib (GOLD)

Dovitinib is an oral TKI that inhibits FGF receptor (FGFR), VEGFR, and PDGFR. In preclinical studies, this agent demonstrated greater antitumor activity when compared to sunitinib and sorafenib (23, 24). Dovitinib has also shown antitumor activity with reported PFS of approximately 5 months in early-phase clinical trials in patients previously treated with VEGF and mTOR inhibitors. For the same setting, median PFS of patients treated with sorafenib ranged between 3.4 and 4.4 months, in different phase II studies (16, 25, 26).

In the phase III GOLD trial, 284 patients with metastatic clear cell RCC who received one previous VEGF-targeted therapy and one previous mTOR inhibitor were randomized to receive either dovitinib 500 mg orally 5-days-on and 2-days-off or sorafenib 400 mg orally twice daily (27). Patients were also stratified based on the standard disease features for advanced RCC studies. With a median follow-up of 11.3 months, there was no difference in the mPFS between both arms (3.7 and 3.6 months for dovinitib and sorafenib groups, respectively; HR 0.86, 95% CI 0.72–1.04; one-sided p = 0.063).

Most common G3/G4 AEs included hypertriglyceridemia (14%), fatigue (10%), HTN (8%), and diarrhea (7%) in the dovitinib group, and HTN (17%), fatigue (8%), dyspnea (7%), and PPE (6%) in the sorafenib group. Percentage of patients who discontinued dovitinib and sorafenib due to AEs is 15% and 12%, respectively. Despite the good tolerability, dovitinib failed to meet the primary endpoint of PFS versus sorafenib in patients pretreated and progressive RCC, and, consequently, has not been approved by FDA for the treatment of advanced RCC.

## Recently approved agents

### Cabozantinib

Cabozantinib is an oral TKI of MET, VEGFRs, and AXL initially tested in a single–arm, phase I study of advanced RCC patients resistant to VEGF and mTOR inhibitors (28) that has now entered the armamentarium of agents for patients with TKI refractory disease. METEOR was a randomized open-label, phase III trial of cabozantinib versus everolimus in advanced RCC patients with progressive disease after VEGFR TKI therapy (Table 2) (29). There was no limit as to the number of prior therapies although the majority of patients (73%) had received only one prior therapy, sunitinib being the most common. All randomized patients (n = 658) received either cabozantinib at 60 mg PO once daily or everolimus at 10 mg daily. The primary endpoint of the trials was PFS. ORR, survival, and safety were secondary endpoints. Similar to other second-line studies, all patients were stratified based on validated risk factor criteria in advanced RCC. The estimated median PFS for patients receiving cabozantinib was 7.4 months compared to 3.8 months for those receiving everolimus. The HR for progression of death while on therapy was higher in the everolimus-treated population (HR 0.58; 95% CI 0.45 to 0.75; P < 0.001). The PFS benefit observed with cabozantinib was observed independent of MSKCC risk-group or first-line treatment received. The ORR was higher in those receiving cabozantinib (21%) compared to those on everolimus (5%) (P < 0.001). More recently, the final OS results were published (30). The median OS was 21.4 months for patients treated with cabozantinib compared with 16.5 months for those who received everolimus (HR 0.66, 95% CI 0.53–0.83, P < 0·001).

**Table 2:** Second-line trials (METEOR/CheckMate 025/Lenvatinib–Everolimus).

	Cabozantinib	Nivolumab	Lenvatinib–Everolimus
**MSKCC risk good/int/poor**	45/42/12	35/49/16	24/37/39
**Comparator**	Everolimus	Everolimus	Everolimus
**Endpoint**	PFS	OS	PFS
**ORR, %**	17	22	35
**PFS, months**	7.4	4.6	12.8
**OS, months**	21.4	25.0	25.5

MSKCC, Memorial Sloan Kettering Cancer Center; ORR, overall response rate; OS, overall survival; PFS, progression-free survival.

Although others have been using this agent in thyroid cancer with no significant safety concerns (31), it is important to point out the safety profile observed on this study. Dose reductions were almost three times more common with cabozantinib compared with everolimus (60% vs. 25%, respectively). In both groups, approximately 10% of patients discontinued therapy due to drug intolerance, and the incidence of >G3 AEs was similar for both drugs (68% and 58%, respectively). Although HTN, diarrhea, and fatigue were the most common G3 AEs observed with cabozantinib, the most common G3 AEs reported with everolimus included anemia, fatigue, and hyperglycemia. Cabozantinib 60 mg per day eventually gained FDA approval in April 2016 and has become a standard of care in the second-line setting (8).

### Nivolumab

Recent findings have demonstrated the importance of the programmed cell-death protein 1 (PD-1) and cytotoxic T-lymphocyte-associated protein 4 (CTLA-4) pathways in immune surveillance. This had led to the rapid development of agents capable of blocking these targets. A number of checkpoint inhibitors including ipilumumab (CTLA-4 inhibitor), atezolizumab (programmed death ligand 1 [PD-L1] inhibitor), and nivolumab and pembrolizumab (PD-1 inhibitors) have been approved for a variety of cancers. These antibodies block PD1 or its ligand PD-L1. Although atezolizumab and pembrolizumab are undergoing clinical development in RCC, nivolumab is the only PD1 inhibitor currently FDA approved in the VEGF TKI refractory RCC space. Nivolumab is a fully human IgG4 PD1 immune checkpoint inhibitor that selectively blocks PD1 expressed in T cells and PD-L1 and PD-L2 expressed in tumor cells and other immune cells (32).

CheckMate-025, a phase III study, compared nivolumab to everolimus in patients with VEGF TKI refractory advanced RCC (Table 2) (9). A total of 821 patients previously treated with one (72%) or two (28%) antiangiogenic drugs were randomized to receive 3 mg/kg IV once every 3 weeks or 10 mg everolimus orally once daily. The primary endpoint was OS, and secondary endpoints included ORR, PFS, and correlation of OS with expression of PD-L1. The median OS was 25.0 months with nivolumab and 19.6 months with everolimus (HR 0.73, 98.5% CI 0.57–0.93; P = 0.002). The ORR was also greater with nivolumab than with everolimus (25% vs. 5%; P = 0.002) but median PFS was similar between the two groups. A subset analysis of the 756 (92%) patients with quantifiable tumor PD-L1 expression showed a significantly higher OS for patients treated with nivolumab, regardless of baseline factors, PD-L1 status, and number of prior therapies (33).

Nivolumab was also generally better tolerated than everolimus. G3/G4 treatment-related AEs occurred in 19% of the patients receiving nivolumab and in 37% of the patients receiving everolimus. The most common AE with nivolumab was fatigue (in 2%), whereas the most common AE with everolimus was anemia (in 8%). Furthermore, nivolumab was also associated with improved QOL compared with everolimus (34). Based on the survival benefit and favorable toxicity profile, the FDA approved nivolumab in November 2015 for the treatment of patients with advanced RCC who have received prior anti-angiogenic therapy (35).

### Lenvatinib–everolimus combination

Recognizing the importance of VEGFR and mTOR pathways in RCC, a synergistic effect with dual inhibition has been suggested in preclinical and phase I and II studies (36). A recent phase II clinical trial studied this combination of the mTORi everolimus with lenvatinib, an inhibitor of VEGFR1–3, Fibroblast Growth Factor Receptor 1–4 (FGFR1–4), Platelet Derived Growth Factor Receptor alpha (PDGFRa), RET, and KIT (Table 2) (37, 38). In this study, Motzer and colleagues randomized 153 patients to receive single-agent lenvatinib 24 mg (n = 52), single-agent everolimus 10 mg (n = 50), or the combination of both agents 18 mg/5 mg (n = 51) (39). The primary endpoint was PFS. Patients were stratified by two factors: hemoglobin (men, ≤130 g/L and >130 g/L; women, ≤115 g/L and >115 g/L) and corrected serum calcium (≥2.5 mmol/L and <2.5 mmol/L).

Patients allocated to lenvatinib plus everolimus were treated for a median of 7.6 months, compared with 7.4 months for those assigned to single-agent lenvatinib and 4.1 months for those who received single-agent everolimus. Median PFS was 14.6 months for lenvatinib plus everolimus and 5.5 months for single-agent everolimus (HR 0.40, 95% CI 0·24–0.68; P < 0.001). Patients treated with single-agent lenvatinib had a median PFS of 7.4 months, which was also longer compared with those treated with single-agent everolimus (HR 0.61, 95% CI 0.38–0.98; P = 0.048). In an updated OS analysis, the median was 25.5 months for lenvatinib/everolimus, compared with 15.4 months for patients treated with everolimus (HR 0.59, 95% CI 0.36–0.96, P = 0.065), which demonstrated a trend towards survival benefit with the experimental combination (40). Overall, ORR was achieved by 43% of the patients allocated combination regimen compared with 27% assigned lenvatinib (ORR 1.6, 95% CI 0.9–2.8; P = 0.10) and 6% who received everolimus (ORR 7.2, 95% CI 2.3–22.5; P < 0.001).

Equally important were the results addressing the safety profile of these drugs. In this study, the most common significant (G3/G4) treatment-related AEs in patients allocated lenvatinib plus everolimus were diarrhea (20%), proteinuria (19%) in those assigned to single-agent lenvatinib, and anemia (12%) in those assigned to single-agent everolimus. Consequently, discontinuation rate secondary to AEs was 24% for patients treated with the combination regimen and single-agent lenvatinib, compared with 7% for patients treated with everolimus. Two deaths were possibly related to study drug, one cerebral hemorrhage in the lenvatinib plus everolimus group and one myocardial infarction with single-agent lenvatinib.

Of note, there is no randomized phase III study confirming the results of this study. Therefore, limitations such as population selection and false-positive rates may had originated biased results (41, 42). Nonetheless, based on the positive results from this phase II study, the combination of lenvatinib and everolimus was recently approved by FDA (May 2016) for the treatment of patients with advanced RCC pretreated with one anti-VEGF therapy (43). A dose finding trial comparing the approved combination dose of lenvatinib and everolimus 18 mg/5 mg with 14 mg/5 mg was mandated by FDA to figure out what is the best combination dose. This trial is planned to open soon.

## Discussion

Metastatic RCC (mRCC) has gained a variety of therapeutic options since the approval of the first VEGF TKI in the mid-2000s. Most recently, the treatment landscape for advanced RCC has drastically changed with results from three randomized clinical trials leading to the approval of nivolumab, cabozantinib, and the combination of lenvatinib/everolimus in previously treated advanced RCC (44). However, as no head-to-head trials exist, physicians are faced with the challenge of choosing the appropriate therapy for an individual patient. As such, clinical decisions are frequently made based on multiple factors including patient comorbidities, disease characteristics, known drug toxicities, and physician experience with different drugs.

Because RCC has joined the list of tumors where immune checkpoint inhibitors have shown to significantly improve OS and the possibility of long-term disease control for a subgroup of patients is real, the favorable side effect profile of these agents makes them a particularly appealing option. In opposition to other drugs, the majority of patients will develop minimal side effects; therefore, nivolumab may be considered for more fragile patients with lower performance status, multiple comorbidities, and organ dysfunction, including renal impairment, otherwise ineligible or borderline eligible for other therapies (45).

Acknowledging that only a subgroup of patients potentially benefits from this class of agents and the absence of a reliable biomarker to help us selecting those patients, one of the questions that will need to be answered is the optimal timing of immunotherapy in RCC. Since its approval, most patients receive nivolumab as second-line agent with the expectation of an improved QOL and the possibility of a long-lasting response to therapy.

Based on Checkmate 025, where patients had received up to three previous antiangiogenic therapies, approximately one-third of the patients were treated in third-line setting, suggesting a benefit later in the course of the disease. On the contrary, multiple clinical trials are ongoing, studying nivolumab and other checkpoint inhibitors in first-line setting, as single agents or in combination with VEGF inhibitors. We anticipate that the results of these studies may help answering this question of best timing for this class of agents (44, 46–48).

Cabozantinib and lenvatinib/everolimus may be two attractive options to consider for patients with rapidly progressive disease or those who are primary refractory to VEGFR TKI-based therapy. Both agents can lead to cytoreduction with more than 80% of patients achieving clinical Partial Response (cPRs) or SD with tumor burden reduction, regardless of performance status, number of prior lines, and metastatic sites (visceral vs. bones) (29, 40, 49, 50). For patients with treated brain metastasis, cabozantinib may also be considered an effective option. Contrary to Checkmate 025 and Len/Eve studies, this subgroup of patients was allowed in the METEOR phase III trial. From a logistical perspective, both cabozantinib and lenvatinib/everolimus are oral therapies and do not require frequent clinic visits for drug administration as required for those receiving nivolumab. Conversely, their toxicity profile constitutes an important limitation for many patients, especially those heavily pretreated. Approximately 10% of patients discontinued cabozantinib due to drug intolerance, and two thirds reported an incidence of >G3 AEs, whereas most patients receiving envatinib/everolimus (89%) reported significant AEs that led to dose reductions or interruptions. Similarly, nearly 20% had significant diarrhea, which led FDA to include a special warning on the drug’s label about the risk of this AE and mandated the investigation team to conduct a dose-finding study with an alternative dose combination (43). To overcome the limitation of toxicity with cabozantinib, there is the option to reduce the dose to 40 mg or even 20 mg per day, as allowed by the research protocol. Many physicians therefore avoid treatment with the approved dose of 60 mg daily and instead treat patients at lower doses, though the loss of efficacy at these doses is not completely known. Beyond the newer agents of nivolumab and cabozantinib, treatment with axitinib remains a less attractive but valid option in the second-line setting particularly with the use of individualized dose titration, which may help to potentiate its clinical benefit (51).

Finally, the role of monotherapy with mTOR inhibitors has become less popular in advanced RCC and should be reserved for patients with molecular genotypes that would predict response to mTOR-based therapy (i.e. TSC1/2 mutations).

Despite the array of approved drugs based on randomized trials, there are still no good data on sequencing strategies to support the use of a particular agent in the second and additional lines of therapy. Similarly, the multiple biomarker candidates from plasma, tumor, and host tissues have failed to identify patients more likely to respond to these therapies (52). PD-L1 and MET staining in both METEOR and Checkmate 025 trials are examples of these challenges (9, 30).

In the absence of biomarkers that can predict response to existing agents, most physicians will define their second- and third-line therapy based on tolerability, AEs, QOL, and survival benefit. Based on randomized phase III data, nivolumab and cabozantinib are likely be the two agents of choice in these clinical scenarios. Nivolumab is a much more attractive option after TKI-based therapy when one looks at its AEs and compared those to the ones reported in the METEOR study. It is also mechanistically different to TKI-based therapy, and its broad utilization and activity across other solid tumors makes it a very attractive treatment alternative in the second-line setting after pazopanib- or sunitinib-based therapy. Others might feel that a rapid response is required especially for those patients who are primary refractory to first-line VEGFR TKI therapy. In this case, it is fair to say that cabozantinib can lead to rapid responses, often in less than 60 days. Main issues, however, remain tolerability as most patients do require dose reductions within the same time frame of 60 days. Although the misconception that checkpoint inhibitors cannot lead to rapid response remains, it is important to note that the median response to nivolumab in Checkmate 025 was 3.5 months. Similarly, best response at 4 months appears to predict for OS benefit as demonstrated in the subset analysis by Motzer et al. (33). As for the combination of lenvatinib/everolimus, the randomized phase II design of the study coupled with concerns for AEs and the logistical complexity of using two different agents at the same time have made it a less attractive therapeutic option in the second- or third-line space. In summary, patients with progressive disease after first-line VEGFR TKIs will go on to receive a checkpoint inhibitor making cabozantinib a third-line agent for the vast majority of RCC patients not eligible for clinical trials.

## Conclusion

With the introduction of different options in the VEGFR TKI refractory setting, choosing the ideal therapy remains a biologic and therapeutic challenge. There are currently no predictive biomarkers that determine the best therapy for the right patient, and little is known about the best sequence of treatments. Future clinical trials with drug combinations, predictive biomarkers, and novel therapeutics will hopefully help resolving some of these challenges.

## Conflict of interest

The authors declare no potential conflicts of interest with respect to research, authorship, and/or publication of this article.
